# Fault Diagnosis of Rotary Machines Using Deep Convolutional Neural Network with Wide Three Axis Vibration Signal Input

**DOI:** 10.3390/s20144017

**Published:** 2020-07-19

**Authors:** Davor Kolar, Dragutin Lisjak, Michał Pająk, Danijel Pavković

**Affiliations:** 1 Faculty of Mechanical Engineering and Naval Architecture, University of Zagreb, Ivana Lučića Street 5, 10002 Zagreb, Croatia; dragutin.lisjak@fsb.hr (D.L.); danijel.pavkovic@fsb.hr (D.P.); 2 Department of Thermal Technology, University of Technology and Humanities in Radom, Stasieckiego Street 54, 26600 Radom, Poland; m.pajak@uthrad.pl

**Keywords:** maintenance, rotary machinery, fault diagnosis, convolutional neural network, classification

## Abstract

Fault diagnosis is considered as an essential task in rotary machinery as possibility of an early detection and diagnosis of the faulty condition can save both time and money. This work presents developed and novel technique for deep-learning-based data-driven fault diagnosis for rotary machinery. The proposed technique input raw three axes accelerometer signal as high definition 1D image into deep learning layers which automatically extract signal features, enabling high classification accuracy. Unlike the researches carried out by other researchers, accelerometer data matrix with dimensions 6400 × 1 × 3 is used as input for convolutional neural network training. Since convolutional neural networks can recognize patterns across input matrix, it is expected that wide input matrix containing vibration data should yield good classification performance. Using convolutional neural networks (CNN) trained model, classification in one of the four classes can be performed. Additionally, number of kernels of CNN is optimized using grid search, as preliminary studies show that alternating number of kernels impacts classification results. This study accomplished the effective classification of different rotary machinery states using convolutional artificial neural network for classification of raw three axis accelerometer signal input.

## 1. Introduction

Rotating machines in general consist of three major parts—a rotor, rolling or journal bearings (fluid or anti-friction bearings), and a foundation. Since rotary machinery usually operates under a tough working environment, it makes it more vulnerable to various types of faults and increase the complexity of fault diagnosis. A failure in rotating machinery results in not only the loss of productivity but also in the delayed delivery of goods and services to customers and may even lead to safety, economic, and environmental problems. Both studies and experience show that faults develop and occur in rotating machines during normal operation. This results in a variety of failures, finally ending up in reduced availability of equipment and higher operating costs. It can be concluded that early fault detection is important, which emphasizes the necessity of maintenance in manufacturing operations. In general, maintenance function is considered as necessary cost in industry. Alternatively, by looking at rotating machinery as a profit center that produces profit only when it is running, it can be concluded that by using modern condition-based maintenance strategy additional net revenue can be generated [1,2,3,4].

**Figure 1:**
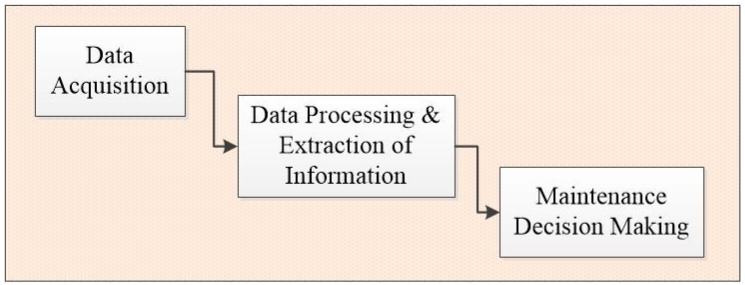
Condition Based Maintenance process.

Nowadays, by using predictive maintenance (i.e., condition-based maintenance) as a maintenance program that recommends maintenance actions based on the processed data collected through condition monitoring [5], maintenance cost and failures can be reduced. Likewise, vibration monitoring is recognized as a leading technique for equipment condition detection and diagnostics. Vibration in any rotating machinery is caused by faults like imbalance, misalignment, crack, etc. Analyzing vibration signature is considered the most powerful predictive maintenance technique [6,7,8] capable of capturing vibrations of the rotating machinery and presenting it in the form of the simple harmonic motion in terms of variation in the amplitude of the vibration signal. While data acquisition as a first step (see Figure 1) of the process is well covered in both research and practice by means of still constantly improving hardware components (sensors and data acquisition systems), the second and third step are currently intensely research-active. In recent years, many techniques for signal processing and extraction of information in fault diagnosis were titled in research, primarily focusing in improving the currently available (traditional) or developing new techniques [9].

Research in prognostic and diagnostic support for decision-making is concerned with the identification of failures and forecasting the remaining useful life of equipment. Although the methods for prognostic and diagnostic support may be similar, the way of their implementation is different: prognosis is based on early failure detection, while diagnosis places greater emphasis on the determination of parameters and failure mode of the already occurring failure. Advancement in technology of measurement equipment and computing together with the increase in the number of data collected reinforces the importance of applying adequate techniques for processing collected data and thus supporting the decision-making process. Various sources categorize decision support approaches in predictive maintenance in a different way, but they can generally be divided into approaches based on physical models and approaches based on historical data. Physical model-based approach presupposes existence or creation of the digital twins of the real system, capable of simulating real behavior of the machines. Creating such models can be a demanding task, considering the possible complexity of the equipment. The development of sensor industry, communication protocols, and Industrial Internet of Things leads to a lower price and greater availability of sensors and data acquisition and processing systems, consequently leading to the greater ability to extract knowledge from these available data. With the increase in the amount of condition data collected, it is possible to create another type of model that describes the system in operation and can provide accurate diagnosis result—data-driven models. They are becoming suitable even for the complex systems and are receiving more and more attention from the researchers and engineers. One of the most used method for data-driven fault diagnosis is machine learning, within which support vector machines [7,10,11,12], and finally artificial neural networks (ANN) algorithms [13,14,15] have been used. It can be noticed that artificial neural networks are often used as classifiers, but as such includes prior definition of the features that need to be extracted from the data collected. Condition-characteristic features definition and extraction has great impact on the result and requires expert knowledge of signal processing techniques. Furthermore, defined features are most often only applicable in that scenario. In recent years, deep learning techniques have achieved huge success in image [16,17,18,19], and medicine engineering [20,21]. Most recently, researchers are beginning to exploit the potentials of deep learning in fault identification and diagnostics, with the aim of reducing or eliminating the shortcomings of shallow ANN architectures. Deep learning stands for class of machine learning techniques specific for its many layers of information processing stages in deep architectures that are exploited for pattern classification and other tasks [22].

The contributions of this paper are summarized as follows:

(1) We propose a multi-channels deep convolutional neural network (MC-DCNN) configuration for rotary machinery state classification, which is used to fuse feature extraction and learning phases of the raw accelerometer data thus eliminating necessity expert knowledge in vibration signal preprocessing. In the first phase of the learning, convolutional layers are used to learn features that are then used as inputs in fully connected layer of the MC-DCNN. Because CNN can learn and then recognize patterns of data that are characteristic for labeled input, wide 1D accelerometer data matrix with dimensions 6400 × 1 × 3 is used as input for convolutional neural network training.
(2) Presented technique is tested on laboratory data in a way that models are trained with different combinations of hyperparameters using grid-search. The comparison of the trained model shows that different hyperparameters combinations has great impact on model performance.
(3) Since convolutional neural networks (CNN) are generally considered as black-boxes, we try to find a physical interpretation of convolutional layers automatic feature extraction with converting learned features in frequency domain by using fast Fourier transform algorithm. By using such an interpretation, it can be seen in which frequency range features are learned for each accelerometer axis. Additionally, we performed activations dimensionality reduction with t-SNE for better understanding how features are learned throughout network.

This paper organizes as follows: In the next section, convolutional neural network used in this study is explored. In Section 3, experimental setup and collected data are explained. Section 4 reveals results of the research. Finally, conclusion is drawn is Section 5.

## 2. Convolutional Neural Network

**Figure 2:**
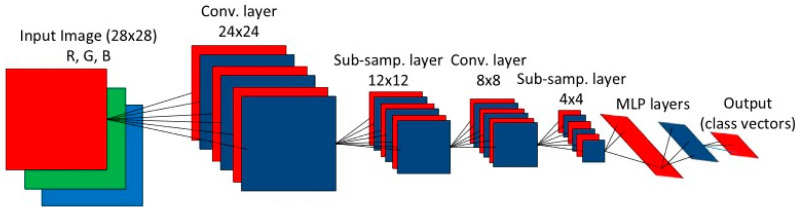
Overview of a conventional 2D convolutional neural networks (CNN) Ince et al. [24] with permission from IEEE.

Convolutional neural networks are biologically inspired feed-forward artificial neural network (ANN) that present a simple model for the mammalian visual cortex, that are proposed by LeCun et al. [23] and now widely used and virtually have become the standard in many object recognition systems in an image or video. The main reason behind such superiority lies in the configuration of CNNs. Convolutional layers use linear kernels, whose parameters are optimized during the training process. Figure 2 illustrates a 2D CNN model with an input layer accepting 28 × 28 pixel images, where image size represents input layer size. Unlike classic ANNs, each neuron of the first hidden layer is not connected with all input layer neurons, yet it is associated with local receptive fields defined by size of the initially defined weight matrix (kernel) and created by sliding the local receptive field over by one neuron for each first hidden layer neuron. This means that all the neurons in the first hidden layer detect the same feature, just at different locations in the input layer. Each convolution layer after the input layer alternates with a sub-sampling layer, which decimates the propagated 2D maps from the neurons of the previous layer. Unlike handcrafted and fixed parameters of the 2D filter kernels, in CNNs they are trained by the back-propagation (BP) algorithm. However, the kernel size and the sub-sampling factor, which are set to 5 and 2, respectively, for illustration purposes in Figure 2, are the two major parameters of the CNN. The input layer is only a passive layer that accepts an input image and assigns its (R, G, B) color channels as the feature maps of its three neurons. With forward propagation over a sufficient number of sub-sampling layers, they are decimated to a scalar at the output of the last sub-sampling layer. The following layers are identical to the hidden layers of an MLP, fully-connected and feed-forward. These so-called fully connected layers end up with the output layer that produces the decision (classification) vector. Convolutional neural networks stand for one of the most effective deep learning architectures and has been applied to fault diagnosis.

Currently, several papers can be found in the field of predictive maintenance that are dealing with the application of deep learning for the detection and diagnosis of equipment conditions.

Janssens et al. [25] analyzed the application of deep learning for automatic feature learning. They used a single layer of a convolutional neural network to learn the characteristics of a vibrating signal, as an example of a bearing failure, and concluded that there is a possibility to apply the technology of convolutional neural networks to learn features. However, the results achieved with an overall classification accuracy of 93.61% proved that there is room for progress.

In some researches, the rotating machinery data is transformed in 2-D image format which is afterwards used for training model. In Do and Chong [26], Chong et al. suggested an approach to extract features from the signal by converting it to 2-D images. Similarly, Wen et al. in [27] investigated another signal-to-2D image conversion as a step to extracting features based on the LeNet architecture. LetNet is one of the first and simplest architectures used in image processing and is a standard template when developing new architectures. They also defined the process of converting a signal to a 2D grayscale image as a way of preprocessing. Further on, Shaheryar et al. [28] explored CNN in fault identification with spectrograms of vibration images previously converted using short time Fourier transform. Specifically, they used a convolutional neural network in combination with an autoencoder network and a fully coupled classification layer to identify the stages of bearing damage on an available dataset. In [29], Hoang and Kang investigated the application of a convolutional neural network to model the stage of bearing damage on an available dataset. They used the raw vibration signal in the time domain from a single uniaxial sensor as input to the model but process it to convert the 1D sensor signal into 2D grayscale images, which they further used for learning. They pointed out that there is no standard method for selecting the correct hyperparameters of the convolution layers, which significantly depends on the learning process. Furthermore, they pointed out that for a computer-intensive learning process it is advisable to implement it on a GPU.

Like Shaheryar, Zhang et al. in their papers [2,30] examined the possibility of applying convolutional neural networks to identify and classify the stages of bearing damage on an available dataset. The test dataset manages to prove that by applying deep structures it is possible to classify the bearing damage stage, even if noise is added to the data in the test set. Furthermore, Shaobo Li et al. [31] presented a new way of using convolutional neural networks in classifying the stages of bearing damage through sensory fusion. They suggest the processing of raw data and the use of the root mean square (RMS) of the frequency spectrum of the signal as an input signal for learning.

In contrast to classification of images, raw signal data can be described as 1D multivariate time series. Most recently 1D CNNs are used for the classification of electrocardiogram (ECG) beats [32] achieving the high performance in terms of both accuracy and speed. Abdeljaber et al. [4] applied 1D CNN in damage detection, while same type of networkin studies of Shao et al. [3], Ince et al. [24] have achieved satisfactory accuracy for fault detection in induction motors. In their study, Zhang et al. [33] introduced end-to-end solution for bearing degradation classification. Further on, Zheng et al. [34] introduced a deep learning framework for multivariate time series classification named multi-channels deep convolutional neural network. In their review article, Lei et al. [35] presented current state and roadmap for machine learning-based fault diagnosis. However, most of the research investigated include one or more signal conversion that in the same time requires an expert to perform.

## 3. Architecture of CNN for Raw Signal Data Input

Most of the data-driven techniques cannot handle raw sensor data hence signal preprocessing in data driven fault identification and diagnosis is of crucial importance. Primarily, data processing aims to extract features of the raw sensor data, that can be used in model training. Extracting and identifying correct features can be difficult and requires expert knowledge. The idea of this study is to use raw time domain accelerometer signal in three axis as 3-channel image input of convolutional neural network. In this study modified multi-channels deep convolutional neural network configuration is used to fuse feature extraction and learning phases of the raw accelerometer data, which can eliminate expert knowledge in vibration signal preprocessing. Multivariate raw signal data is divided into univariate in a way that each channel (signal axis) presents input in a feature learning stage.

**Figure 3:**
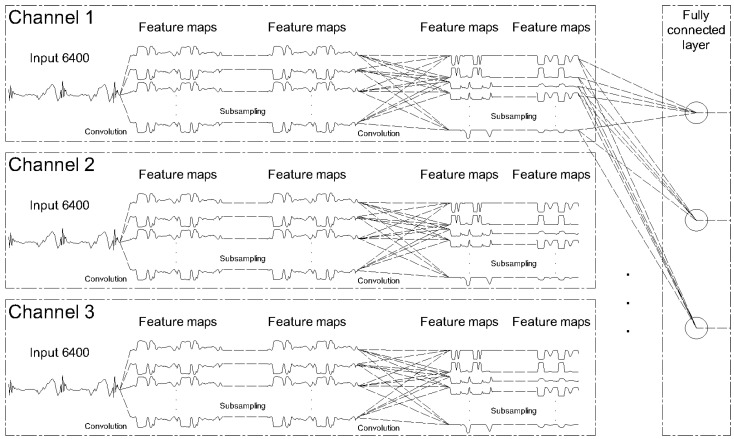
Two-staged modified MC-DCNN architecture with three channels input.

For each channel 2-stage feature learning is done and after that learned features are concatenated in a fully connected layer, as it can be seen in Figure 3. Multi-channels deep convolutional neural network (MC-DCNN) consists of two main parts. First is a feature extractor, that is used for automatic learning features from raw data and the other is trainable fully connected multi-layer perceptron (MLP), which performs classification based on the features learned in the first stage. Feature extractor is composed of multiple similar stages made up of three cascading layers: filter layer, activation layer, and pooling layer. The inputs and outputs of each layer are called feature maps. Specifically, modified 2-stages MC-DCNN for failure classification is developed. Input signal consists of three channels and length of each input is 6400. The input (i.e., the univariate time series) is fed into a 2-stages feature extractor, which learns hierarchical features through filter (kernel), activation, and pooling (sub-sampling) layers. The MC-DCNN contains two convolutional layers with alternating kernel number, each followed by max pooling layer, finally ending with fully connected layer, output unit activation function, and classification layer. The output unit activation function is the softmax function:

(1) $$y_{r}(x)=\frac{\exp(a_{r}(x))}{\sum_{j=1}^{k}\exp(a_{j}(x))}$$

## 4. Mini-Batch Stochastic Gradient Descent with Momentum-Based Learning

The loss function for previously observed CNN is defined as cross entropy function:

(2) $$E=-\sum_{i=1}^{n}\sum_{j=1}^{k}t_{ij}\ln y_{j}(x_{i},\theta )$$

where *θ* is the parameter vector, *t_ij_* is the indicator in which the *i*-th sample belongs to the *j*-th class, and *y_j_*(*x_i_*, *θ*) is the output for sample *i*, respectively. The output *y_j_*(*x_i_*, *θ*) can be interpreted as the probability that the network associates *i*-th input with class *j*, that is *P*(*t_j_* = 1| *x_i_*). A full cycle of parameter updating procedure includes three cascaded phases Bouvrie [36]: feed forward pass, back propagation pass, and the gradient applied. Widely used mini-batch gradient-based back propagation with momentum is used to minimize the loss function Lecun et al. [37]. In Keskar et al. [38] authors found that models respond better during testing when trained on smaller batches and then update parameters. The weight *wl* was updated as described by the following equations:

(3) $$w_{ij}^{l}=w_{ij}^{l}+△w_{ij}^{l}$$

(4) $$w_{ij}^{l}=momentum\cdot △w_{ij}^{l}-\varepsilon \cdot w_{ij}^{l}-\varepsilon \cdot \frac{\partial E}{\partial w_{ij}^{l}}$$

where *w*^*l*^ = *momentum Ow^l^ ε w^l^ ε·^∂E^* represents the weight between *x^l−1^* and *x^l^*. Further on, *Ow^l^* denotes the gradient of *w^l^* and *ε* denotes the learning rate. *Momentum* value is set to 0.7 and learning rate to 0.01 with mini-batch size of 128, respectively. Additionally, learning rate is specified to multiply by factor 0.1 after each 10 epochs. Both initialization and the momentum play an important role in convolutional neural networks performance, hence future research in this field is necessary. Additionally, learning of the network parameters will stop after 10 consecutive epochs improvement, i.e., validation loss reduction.

## 5. Experimental Setup

**Figure 4:**
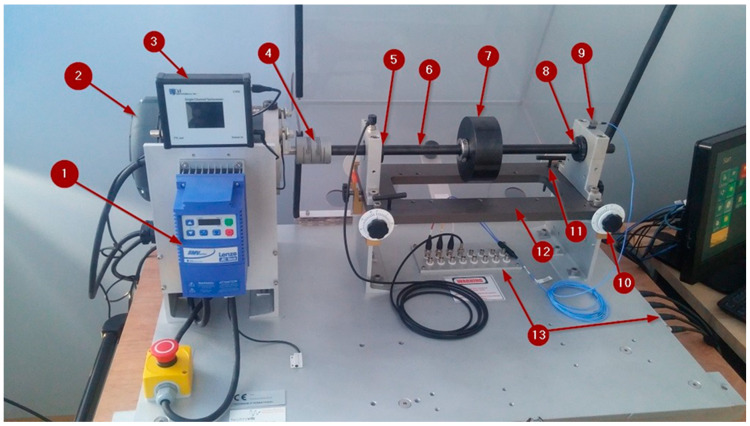
Fault simulator. (1) Frequency drive Lenze SMVector; (2) motor; (3) tachometer display; (4) clutch; (5) front-end bearing; (6) main shaft; (7) load; (8) back-end bearing; (9) three-axis IEPE accelerometer; (10) horizontal axis alignment screw; (11) vertical axis alignment screw; (12) base; (13) BNC connectors.

The developed CNN technique is tested on experimental data collected in Laboratory for Maintenance of University of Zagreb, Faculty of Mechanical Engineering and Naval Architecture. In this study, the vibration signals acquired from a machine fault simulator are used. A SpectraQuest variable speed machinery fault simulator (MFS) was used to generate both normal operation and faulty condition data. The system (illustrated in Figure 4) consist of a 0.75 kW variable speed motor driving a shaft-rotor component via coupling supported with two sets of ball bearings. This type of simulator can be used for performing various controlled experiments on a device that emulates real world machinery. Different modules representing different rotary machinery states can be mounted on a device. Examples of fault states that can be simulated are rotor faults (cocked, eccentric, and disbalanced rotors), AC motor faults, bearing and bearing housing faults, as well as faults generated by multi-belt drives, worn gearbox parts, fan, compressor, and pumps vibrations etc. Current configuration of MFS is outfitted with three-axis accelerometer and a tachometer, that are connected to a National Instruments DAQ System.

Although there are several other types of sensors that can serve as data sources for condition monitoring, accelerometers have been selected and used in this study because of the fact that they are widely used in practice. Three-axis PCB Piezotronics 356B21 IEPE type accelerometer is mounted on the bearing housing on the shaft side opposite of the motor position. The sampling frequency is set to 6.4 kHz, while revolving speed during the experiment is set to 1500 r/min. Vibration signals in three directions are acquired when the system operates under three different conditions. Each acquired sample of 6400 data points is stored as data set representing state. Vibration signals under four different working conditions are used in this study, and they are divided into training and testing data sets separately, which are randomized before being used in training and testing the model. The descriptions of them are listed in Table 1.

## 6. Description

Convolutional neural network training is done on GPU of our machine learning platform.

12,000 data sets have been collected to train and test the convolutional neural network data-driven model for failure classification. Table 2 illustrates the data composition of collected samples. From all the samples, 70% of the data is used for training and validation during training while rest 30% is used for testing the model. 10% of training data is used for validation during training. The samples for training, testing, and validation during the experiment were selected randomly.

## 7. Results

In this section, we will discuss the diagnosis accuracy of the proposed technique for fault classification. The CNN structure on this study contains two alternating convolutional and pooling layers with one fully connected layer followed by softmax and classification layer. First convolution layer uses wide kernel (31), while second kernel size is smaller (4). By using such a combination of kernels, all 6400 univariate time series samples spread across three channels are used for feature learning. The parameters of each layer are presented in Table 3. First convolutional layer output consists of k1 feature maps calculated using k1 number of kernels, that are translated into second layer inputs. Further on, by using k2 number of kernels, k2 feature maps of second convolution layer are calculated. Sub-sampled feature maps of second convolution layer are used as fully connected layer input.

CNN_k1–k2 denotes that there are k1 number of kernels in convolutional layer 1 and k2 number of kernels in convolutional layer 2. There are nine models with alternating number of kernels in first and second convolutional layer. Each CNN model training runs 100 times, and mean, minimum, maximum, and standard deviation of the classification accuracy are the results measure terms presented in Table 4.

**Figure 5:**
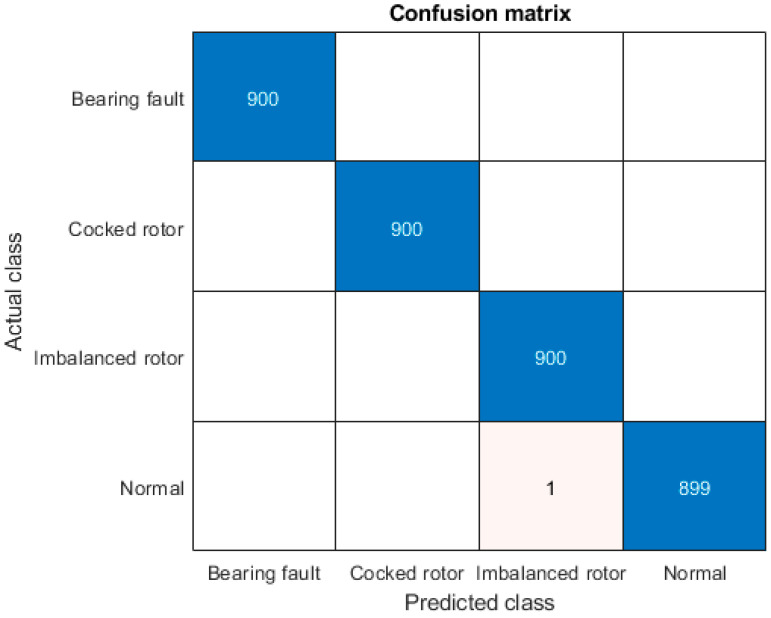
Confusion matrix of CNN_24–48.

From the results, best average accuracy achieves CNN_24–48 with average accuracy of 99.93% and maximum achieved accuracy of 99.97% with standard deviation of 0.0506%. Confusion matrix for best CNN_24–48 (bolded in Table 4) has been shown in Figure 5.

The best maximum accuracy is 100% achieved by CNN_24–16, while in the same time this network has also produced the lowest accuracy of 99.64%, respectively. The lowest mean accuracy achieved CNN_8–48. Overall, all networks have mean accuracy equal to or greater than 99.80%. Additionally, networks with higher number of kernels in first layer gained slightly better performance.

**Figure 6:**
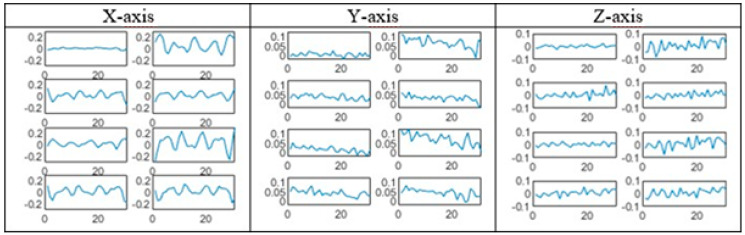
Kernels of first convolutional layer.

**Figure 7:**
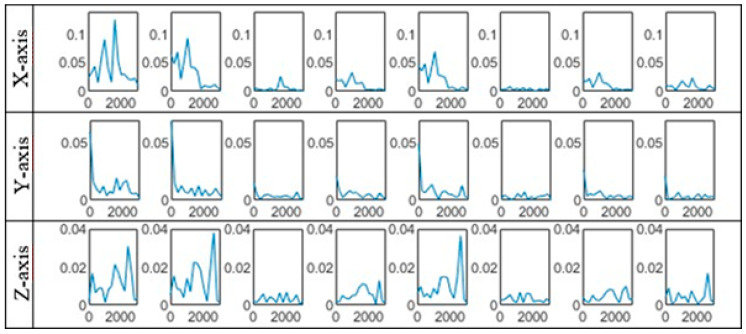
Frequency domain representations of kernels of first convolutional layer.

Model output also contains information about classification uncertainty. Since it covers four different machine states, all unknown states i.e., states not covered in presented model are handled by decision support application which suggests model retraining with new data in case of high class uncertainty. Although CNN-s are widely presented as black-box solutions and it is somewhat hard to understand the inner operating mechanisms, activations can be visualized. For the CNN_8–16 (bolded in Table 4), we plot kernels of the first and second convolutional layer for all three axes. Both Figure 6 and Figure 7 give us better insights of features that are learned in first convolutional layer. Although time-domain kernels are physically understandable, better visualization can be done by implementing fast Fourier transformation. Figure 7 presents first convolutional layer kernels learned for each axis of input signal. It is noticeable that most of the features learned for *X*-axis takes place in middle frequency range, *Y*-axis in low and medium range, while *Z*-axis features are extracted from medium to high frequencies. If compared to signal processing techniques, it can be concluded that first convolutional layer features present efficient frequency cut-off filters.

**Figure 8:**
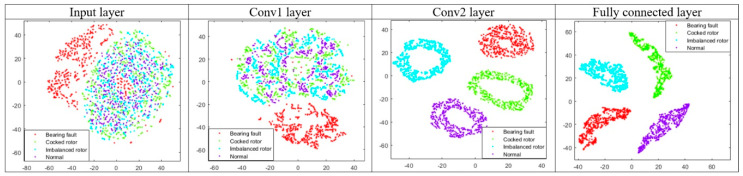
t-SNE activations representations.

Further on, distribution of all test samples extracted from input signal, each convolution layer and fully connected layer for the CNN_24–48 (bolded in Table 4) are given in Figure 8. Visualization is done by t-SNE van der Maaten and Hinton [39]. By looking at the input layer through convolutions, it can be clearly seen that activations computed by learned kernels in high dimensional space can be successfully clustered via t-SNE in clusters representing classes in two dimensions as evolving to the fully connected layer.

## 8. Conclusions

This study proposes a new CNN-based fault diagnosis technique. The main contribution of this study is developing an algorithm that inputs wide raw three-axis accelerometer signal as 1D matrix into features extractor part of convolutional neural network, that consequently automatically extracts features and enables classification. When compared to traditional data-driven fault diagnosis, the omission of the need for manual extraction of features can be highlighted as the main advantage. Additionally, wide input signal used in this study provides full potential of CNN learning process since raw signal is available, while also gaining high classification accuracy.

Different combinations of number of kernels in first and second convolutional layer has been investigated in order to find near-optimal parameters. In order to potentiate physical interpretation of the obtained models, graphical interpretations of the learned kernels and activation has been done. Results show potential of the proposed CNN technique in the data-driven fault diagnosis field, especially since vibration signals from three axis accelerometer enters model without any time-consuming manual feature extraction.

Networks trained with higher number of kernels in first layer gained slightly better performance, while the best maximum accuracy is 100% achieved by CNN_24–16.

Limitations of developed technique can be considered in the form of applications on real rotary machinery. Common faulty conditions must be detected and labeled for training purposes, as previously not learned faults could be misclassified. Likewise, additional testing of proposed technique on different types of failures and varying load as well as on known datasets is essential for performance comparison. Further on, selecting optimal hyperparameters is still a challenge. Additionally, other signals that could be used in combination with vibration data to represent machine state, such as current, acoustics emission, stray flux, etc., were not included in this research. Finally, training process of developed MC-DCNN is time demanding and using GPU hardware is highly advisable. Taking that into account, future work will be based on additional testing of the technique, including as well as on doing research about hyperparameter optimization.

## Figures and Tables

**Table 1:** Simulated fault conditions.

No	Condition	Description
1	Normal State	Machine is running without simulated fault
2	Debalanced Rotor	Machine is running with simulated fault of imbalance on main shaft
3	Cocked Rotor	Fault is simulated by adding cocked rotor on main shaft
4	Bearing Fault	Machine is running with bearing outer race fault

**Table 2:** Composition of collected samples for fault classification.

12,000 datasets collected (76,800,000 data points)	3000 samples collected in normal working condition	2100 samples for training and validation during training (stochastic)
		900 samples for testing (stochastic)
	3000 samples collected in failure type 1: main shaft imbalance	2100 samples for training and validation during training (stochastic)
		900 samples for testing (stochastic)
	3000 samples collected in failure type 2: Cocked rotor	2100 samples for training and validation during training (stochastic)
		900 samples for testing (stochastic)
	3000 samples collected in failure type 3: Bearing fault	2100 samples for training and validation during training (stochastic)
		900 samples for testing (stochastic)

**Table 3:** Convolutional neural network parameters.

Layer	Size and Parameters
Input Layer	Input signal: [6400 × 1 × 3]
Convolutional Layer 1	k1 kernels: [31 × 1 × 3] Layer output size: 6370 × 1 × k1
Activation Layer 1	Rectifier Linear Unit (ReLU)
Pooling Layer 1	Max pooling [2 × 1] Layer size: 3185 × 1 × k1 Stride = 2
Convolutional Layer 2	k2 kernels [4 × 1 × 16] Layer size: 3182 × 1 × k2
Activation Layer 2	ReLU
Pooling Layer 2	Max pooling [2 × 1] Layer size: 1591 × 1 × k2 Stride = 2 Fully connected layer
Fully Connected Layer	Size: 4
Softmax	
Output Layer	Classes

**Table 4:** Results of CNN models with different number of kernels.

CNN	Mean	StDev	Max	Min
CNN_8–16	99.86%	0.0700%	**99.97%**	99.78%
CNN_8–32	99.81%	0.0666%	99.89%	99.70%
CNN_8–48	99.80%	0.0448%	99.89%	99.72%
CNN_16–16	99.86%	0.0275%	99.89%	99.81%
CNN_16–32	99.84%	0.0492%	99.89%	99.75%
CNN_16–48	99.87%	0.0637%	99.97%	99.81%
CNN_24–16	99.86%	0.1036%	100.00%	99.64%
CNN_24–32	99.86%	0.0369%	99.92%	99.81%
CNN_24–48	99.93%	0.0506%	**99.97%**	**99.83%**
